# Sacral Neuromodulation in a Pregnant Patient With Fowler's Syndrome: A Case Report

**DOI:** 10.7759/cureus.11796

**Published:** 2020-11-30

**Authors:** Yahia Alghazwani, Mohammad A Alghafees, Omar Alfraidi, Rakan Aldarrab

**Affiliations:** 1 Urology, National Guard Hospital, Riyadh, SAU; 2 Medicine, King Saud Bin Abdulaziz University for Health Sciences, Riyadh, SAU

**Keywords:** sacral neuromodulation, fowler's syndrome, urogynecology, ksa:kingdom of saudi arabia

## Abstract

Fowler's syndrome (FS) is a condition in which females face chronic urinary retention with abnormal electromyography (EMG) findings in the absence of structural anomalies. A sacral neuromodulation (SNM) device that restores urinary discharge is often used for treatment. It is advised to turn the device off during pregnancy. This is a case report of a 37-year-old pregnant female suffering from FS. The patient was on SNM and underwent two uneventful pregnancies despite the device being kept on throughout both pregnancies. There were no complications, and a healthy term baby was born on both occasions.

## Introduction

Chronic urinary retention, albeit rare, can be very debilitating when present. Fowler's syndrome (FS) usually arises post-childbirth or following surgery. First described in 1985, FS consists of urinary retention due to the urethral sphincter's failure to relax and allow voiding [1].

Due to difficulty in emptying the bladder adequately and the risk of recurrent urinary tract infection (UTI) and its associated fetal and maternal morbidity, FS has significant implications during pregnancy. Despite the absence of any neurological or structural disorders, in patients suffering from FS, there is a high post-void residual volume, the resting urethral pressure is increased, and the urethral electromyography (EMG) is abnormal, as shown by urodynamic studies [2]. The only successful treatment for FS over the last two decades is sacral neuromodulation (SNM) [3]. 

Neuromodulation uses electrical stimulation and chemical agents to modulate neural activity. Spinal cord stimulation (SCS) applies the gate control theory in which the ascending nerve traffic at the segmental level is inhibited by the activation of afferent fibers in the spinal cord. Although most patients suffering from FS are of reproductive age, the effects of SNM on pregnancy and vice versa have not been determined, and the medical literature has conflicting reports regarding the safe use of SNM during pregnancy.

It is rare to use SNM during pregnancy. We present the case of a 37-year-old pregnant female suffering from FS who used SNM throughout her pregnancy and reported no problems.

## Case presentation

In 2017, a 37-year-old patient in the first trimester of her pregnancy presented to the urology clinic with urinary retention. Her initial laboratory workup was normal. She had been diagnosed with FS and was on SNM since 2015. The device was turned off, and her condition was treated conservativelyl; her condition did not improve. Further investigation showed electrode displacement, which is not an uncommon occurrence during pregnancy. She was taken to the operation theater for implant readjustment under general anesthesia. The old device was removed, and a new space was created for the implant, and the device was inserted. After the successful implantation, the patient was discharged with a three-week follow up in which no problems were accessed. The patient responded well to SNM and was closely monitored for any adverse fetomaternal outcomes; none were detected, and a healthy baby was delivered.

Two years later, in 2019, the patient in the second trimester of her pregnancy presented again with urinary retention, leg pain, and surgical site pain. Tenderness and swelling were found at the surgical site upon physical examination. The SNM device was switched off, and it was decided to conduct implant readjustment under general anesthesia once again. After opening at the previous incision, a pocket of pus was removed, and the site was irrigated with gentamycin. The device was then repositioned. The wound was closed, and the device was turned on again. The patient was discharged from the hospital after completion of the procedure and was followed up until delivery. Once again, the pregnancy was uneventful, and no adverse fetomaternal outcomes were reported.

## Discussion

Fowler's syndrome was first identified in 1985 as painless urinary retention in young women and was associated with abnormal urethral sphincter EMG and polycystic ovaries. While the full etiology of the disorder is unknown, the current hypothesis proposes that this disorder is a consequence of hormonally sensitive channelopathy that involuntarily causes a sustained contraction of the striated urethral sphincter. Consequently, the detrusor contractions are inhibited, and the desire to void is reduced [4]. The only successful treatment option for FS is SNM.

Due to the conflicting literature reports, there is no clear evidence that concludes the safety of SNM during pregnancy. Consequently, the manufacturers advise turning the SNM device off as soon as pregnancy is detected, and clinicians typically deactivate the SNM devices during pregnancy in patients suffering from FS [5]. However, medical literature does not offer strong evidence that supports this approach. Neuromodulation, in fact, is extensively used to treat problems such as nausea, vomiting, and placental insufficiency during pregnancy [6]. Since bladder dysfunction in women of reproductive age is a rare phenomenon, our understanding of the effects of SNM on pregnancy is limited. The use of SNM device in pregnancy is often contraindicated by clinicians because of the theoretical risk of a teratogenic effect on the fetus as well as the risk of premature birth as it is possible that the uterus and the bladder may share the same nerve roots [5].

In the case presented, the SNM device was readjusted and kept switched on during both pregnancies in the same patient with no observed teratogenic effects on the fetuses. The same was found by Khunda et al. who also reported no adverse fetomaternal outcomes in pregnant FS patients who used SNM [7]. Some clinical cases and two series of women (n = 5 and n = 13) who were on SNM and became pregnant post-implantation have been reported [7-10]. A study recently done by Yaiesh et al. also reported no adverse fetomaternal outcomes in women kept on SNM during their pregnancy [11]. Another local case report also noted no remarkable effects of using SNM during pregnancy [12].

We are yet to find any reports of complications during pregnancy, labor, and delivery, or any hindrances in the development of children of mothers who were on SNM during pregnancy and lactation. To date, the studies conducted on pregnant women with SNM and on animals do not show altered fetal development due to the use of electrical stimulation [13]. We hope that our case adds more evidence to the safety of SNM during pregnancy and is a ray of hope for those suffering from FS who wish to become pregnant as well for the clinicians managing such patients.

## Conclusions

We did not note any adverse effects of the use of SNM on the fetus, the mother, or the device. Large-scale studies are needed to further investigate the effects of neuromodulation in pregnant females suffering from FS.
